# Bevacizumab and risk of intracranial hemorrhage in patients with brain metastases: a meta-analysis

**DOI:** 10.1007/s11060-017-2693-4

**Published:** 2017-11-23

**Authors:** Lin Yang, Chuan-Jie Chen, Xiao-Ling Guo, Xiao-Cui Wu, Bo-Jie Lv, Hong-Li Wang, Zhi Guo, Xiang-Yang Zhao

**Affiliations:** 1 Department of Oncology, 266 Hospital, PLA, Pu-Ning Road, Shuangqiao District, Chengde, 067000 China; 2 Department of Orthopedics, Chengde Central Hospital, Second Affiliated Hospital of Chengde Medical University, 22 Xi-Da Street, Shuangqiao District, Chengde, 067000 China; 3 Department of Neurology, 266 Hospital, PLA, Pu-Ning Road, Shuangqiao District, Chengde, 067000 China; 4 Department of Scientific Research, 266 Hospital, PLA, Pu-Ning Road, Shuangqiao District, Chengde, 067000 China; 5 Department of Oncology, Shanxi Provincial Hospital of Traditional Chinese Medicine, 16 Bing-Zhou-Xi Street, Yingze District, Taiyuan, 030001 China; 6 Department of Surgical Oncology, 266 Hospital, PLA, Pu-Ning Road, Shuangqiao District, Chengde, 067000 China

**Keywords:** Bevacizumab, Brain metastases, Intracranial hemorrhage, Meta-analysis

## Abstract

Administration of bevacizumab to patients with brain metastases (BM) is controversial due to concerns about the increased risk of intracranial hemorrhage (ICH). This meta-analysis assessed whether the risk of ICH increases in BM patients receiving treatments that contain bevacizumab versus without. PubMed, Embase, Cochrane Library and annual meeting abstracts of the American Society of Clinical Oncology up to 13 November 2016 were searched for studies that referred to ICH complications due to bevacizumab in patients with BM. Eight studies involving 8713 patients were included in this analysis. Compared with the control arm without bevacizumab, the bevacizumab treatment arm did not exhibit a significant increase in ICH [odds ratio (OR) 1.20; 95% confidence intervals (CI) 0.69–2.09; P = 0.53]. Subgroup analyses with retrospective studies showed a similar result, although subgroup analyses with prospective studies failed. This meta-analysis revealed that bevacizumab does not significantly increase the risk of ICH in solid tumor patients with BM.

## Introduction

Brain metastases (BM) are the most common intracranial tumors in adult patients [1] and occur in up to 40% of adult cancer patients [2]. The prognosis of patients with BM is poor, with median overall survival ranging from weeks to months in untreated patients [3]. In adults, BM generally originate from primary lung (40–50%), breast (15–25%), renal, gastrointestinal tract tumors (4–6%) and melanoma (5–20%) [4]. The incidence of BM depends on the tumor type and molecular subtype [5], with melanoma having the highest incidence (approximately 50%) [6]. The incidence of BM is rising for many reason, e.g., increasing occurrence of tumors prone to metastasis to the brain such as lung cancer, wide utilization of powerful imaging technologies such as magnetic resonance imaging during upfront staging and follow-up, longer survival of cancer patients due to earlier detection and better treatment, and the advent of novel therapeutic compounds with good anti-neoplastic activity but inadequate penetration through the blood–brain barrier [5, 7].

Bevacizumab is a recombinant, humanized monoclonal antibody targeting vascular endothelial growth factor (VEGF), which is a key factor associated with tumor angiogenesis and growth [8, 9]. Bevacizumab was proven effective for diverse solid tumors, including metastatic breast cancer [10], non-squamous non-small cell lung cancer (NSCLC) [11, 12], colorectal cancer [13], renal cell carcinoma [14], and recurrent glioblastoma [15]. Until recently, patients with central nervous system (CNS) metastases have been routinely excluded from bevacizumab-containing clinical trials, following a single case in which a severe intracranial hemorrhage (ICH) from a previously undiagnosed brain metastasis was observed in a patient with hepatocellular carcinoma (HCC) during a phase I study of bevacizumab in 1997 [16]. The potential risk of ICH precludes the use of bevacizumab in solid tumor patients with BM [17]. Although several reports have recently concluded that there is no increased risk of ICH in BM patients receiving bevacizumab treatment [8, 18–21], application of bevacizumab in such patients remains controversial. To date, no meta-analysis of association between bevacizumab and ICH risk in patients with BM has been reported. In this study, we performed a meta-analysis to assess whether BM patients receiving treatments containing bevacizumab have a higher risk of ICH than patients receiving treatments without bevacizumab.

## Methods

### Eligibility criteria

Studies that met the following criteria were included: (1) Subjects were solid tumor patients with BM. (2) Experimental arm patients received chemotherapy/targeted therapy with bevacizumab, and control arm patients received the same chemotherapy/targeted therapy with or without placebo. (3) Data were available for ICH.

### Literature search strategy

PubMed, Embase and Cochrane Library were comprehensively searched for studies that referred to ICH complications of bevacizumab in solid tumor patients with BM (data cutoff date: 13 November 2016). The annual meeting abstracts of the American Society of Clinical Oncology were also searched from inception of the database to June 2016. The following search terms were used: ‘adverse effect’, ‘AE’, ‘safety’, ‘toxic*’, ‘side effect’, ‘bleed*’, ‘hemorrhag*’, ‘haemorrhag*’, ‘toleren*’, ‘cerebrovascular event’, ‘complication’, ‘bevacizumab’, ‘avastin’, ‘cancer’, ‘tumor’, ‘carcinoma’ and ‘neoplasm’. The search strategy is shown in Table 1. The search was limited to English publications in human subjects.

**Table 1:** Search strategy

Search	Terms
#1	Bevacizumab OR avastin
#2	Adverse effect OR AE OR safety OR toxic* OR side effect OR bleed* OR hemorrhag* OR haemorrhag* OR toleren* OR cerebrovascular event OR complication
#3	Cancer OR tumor OR carcinoma OR neoplasm
#4	#1 AND #2 AND #3

### Study selection

Two reviewers independently performed the initial search, deleted duplicate records, reviewed the titles and abstracts for relevance, and identified each as exclude or requiring further assessment. If deemed necessary, the full text of the article was retrieved and reviewed in detail to identify eligible studies according to the predefined inclusion criteria. Discrepancies were resolved by consensus.

### Data extraction

Two reviewers independently abstracted data, including the name of the first author, publication year, study design, indication, sample size of each arm, number of patients experiencing ICH in each arm, treatment regimens, bevacizumab treatment time, bevacizumab dose, BM status when patients enrolled in the primary study, and evaluation criteria for ICH. Again, discrepancies were resolved by consensus.

### Quality assessment

Two reviewers independently evaluated the methodological quality of included studies according to the Newcastle–Ottawa Scale (NOS) [22]. The reviewers resolved disagreement by discussion.

### Statistical analysis

Data were analyzed using RevMan 5 (http://ims.cochrance.org/revman/download). Differences were expressed as odds ratios (ORs) with 95% confidence intervals (CIs) for dichotomous outcomes. Heterogeneity across the included studies was evaluated by the Cochrane’s Q-test I² statistic. P > 0.1 and I^2^ < 50% indicated a lack of inter-study heterogeneity; P < 0.1 and I^2^ > 50% indicated that the studies were heterogeneous [23], and we explored the causes of heterogeneity by subgroup, sensitivity, and other analyses. A fixed-effects model (Peto method) was used regardless of heterogeneity because ICH was a rare event in BM patients receiving medical therapy with or without bevacizumab (events with incidence < 1% were defined as rare events) [24]. Publication and selection bias were investigated through funnel plots. A two-sided P value < 0.05 was considered statistically significant.

## Results

### Study identification and selection

**Fig. 1:**
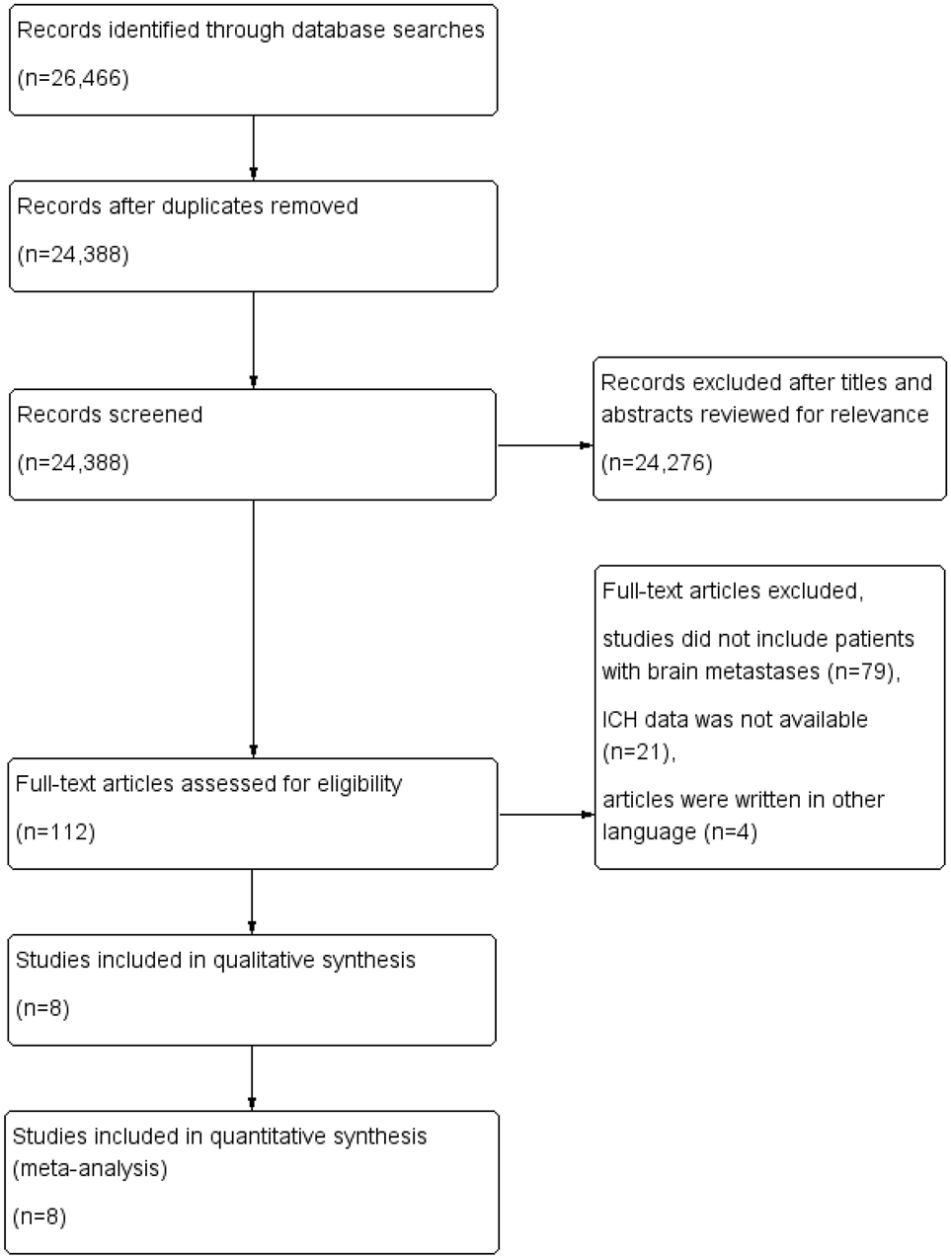
Selection process for the studies included in the meta-analysis

Using our search strategy, a total of 26,466 records were retrieved from the initial database search. After excluding duplicate articles, 24,388 records remained. After a simple reading of the titles and abstracts of the articles, 24,276 records were removed, including articles not potentially relevant to the analysis, articles of primary CNS malignancies, review articles, meta-analyses, case reports or case series, studies without control arms, studies with both arms containing bevacizumab, studies with different regimens in different arms except bevacizumab or placebo, and studies containing other anti-VEGF drugs except bevacizumab. The remaining 112 full-text articles were reviewed in detail, and 104 of them were also removed because the studies did not include patients with BM, articles were written in another language, or the data for ICH was not available. Finally, a total of eight studies were included in this meta-analysis [18, 21, 25–30]. The selection process is shown in Fig. 1.

### Study characteristics

The key characteristics of the included studies are summarized in Table 2. Eight studies involving 8713 patients were included in this analysis. Six studies were retrospective, and two were prospective. 8053 of the 8713 patients were included in the experimental group receiving bevacizumab-containing treatment, and 660 were included in the control group receiving treatment without bevacizumab. Five of the eight included studies reported the dose of bevacizumab in bevacizumab-treated patients. Four out of eight studies had control arms in which patients received placebo. The high- and all-grade ICH events were not differentiated when the study data were abstracted because very few studies reported all-grade or low-grade (grade 1–2) ICH.

**Table 2:** The characteristics of included studies

Study	Design	Sample size		Intervention		Indication	Treatment time of bev	Treated or untreated BM when patients enrolled in primary study	Symptomatic or asymptomatic BM when patients enrolled in primary study	Evaluation criteria for ICH
		Treatment group	Control group	Treatment group	Control group					
Archer et al. [18]	Retrospective	24	26	Bev (15 mg/kg/3 w) + carboplatin + paclitaxel; bev (7.5/15 mg/kg/3 w) + cisplatin + gemcitabine	Carboplatin + paclitaxel, placebo + cisplatin + gemcitabine	Non-squamous NSCLC	First line	Untreated	Symptomatic	NCI CTCAE v. 2.0/3.0
Akerley et al. [25]	Prospective	85	6	Bev (15 mg/kg/3 w) + platinum-based doublet therapy or erlotinib; Bev (15 mg/kg/3 w) + single-agent chemo or erlotinib; Bev + chemo followed by Bev + erlotinib/placebo	Non-bev treatment	NSCLC	First/second line	Treated	Asymptomatic	NCI CTCAE v. 3.0
Oh et al. [29]	Retrospective	179	6495	Bev-based treatment	Treatment without bev	NS	NS	Partly treated	NS	NS
Besse et al. [21]	Retrospective	91	96	Bev (7.5/15 mg/kg/3 w, 5/10 mg/kg/2 w) + chemo/ IFN	Chemo/IFN with or without placebo	Non-squamous NSCLC, RCC, Pancreatic cancer, BC, CRC	First line mostly	Untreated	NS	NCI CTCAE
Dirix et al. [26]	Retrospective	14	10	Bev (7.5/15 mg/kg/3 w) + docetaxel	Placebo + docetaxel	BC	First line	Untreated	NS	NCI CTCAE, v. 3.0
Herbst et al. [27]	Prospective	38	30	Bev (15 mg/kg/3 w) + erlotinib	Placebo + erlotinib	NSCLC	Second line	Treated	Asymptomatic	NCI CTCAE, v. 3.0
Khasraw et al. [28]	Retrospective	112	867	Treatment with Bev	Treatment without Bev	Ovarian cancer, NSCLC, Colon, Sarcoma	NS	Partly treated	NS	NS
Tang et al. [30]	Retrospective	117	523	Bev + chemo	Chemo only	NSCLC	NS	Treated	NS	WHO criteria

*NSCLC* non-small-cell lung cancer, *bev* bevacizumab, *NCI CTCAE* National Cancer Institute Common Toxicity Criteria for Adverse Events, *chemo* chemotherapy, *NS* not specified, *RCC* renal cell carcinoma, *BC* breast cancer, *CRC* colorectal cancer, *WHO* World Health Organization

### Quality assessment

The NOS results are summarized in Table 3. Among the six retrospective studies, one received eight stars, three received seven stars, and two received six stars. One of two prospective studies received nine stars, and the other received eight stars.

**Table 3:** Results of quality assessment for studies using NOS

Study	Study design	Selection	Comparability	Exposure	Outcome
Oh et al. [29]	Case–control	**^ab^	*^c^	***	
Archer et al. [18]	Case–control	***^b^	*^c^	***	
Besse et al. [21]	Case–control	**^ab^	**	***	
Dirix et al. [26]	Case–control	***^b^	*^c^	***	
Khasraw et al. [28]	Case–control	**^ab^	*^c^	***	
Tang et al. [30]	Case–control	***^b^	**	***	
Akerley et al. [25]	Cohort	****	**		***
Herbst et al. [27]	Cohort	****	*^c^		***

Reasons for lost stars: ^a^the case was defined by record linkage; ^b^hospital controls were selected; ^c^there was no description of whether the study controlled for any additional factor, such as gender or age

### Risk of ICH

**Fig. 2:**
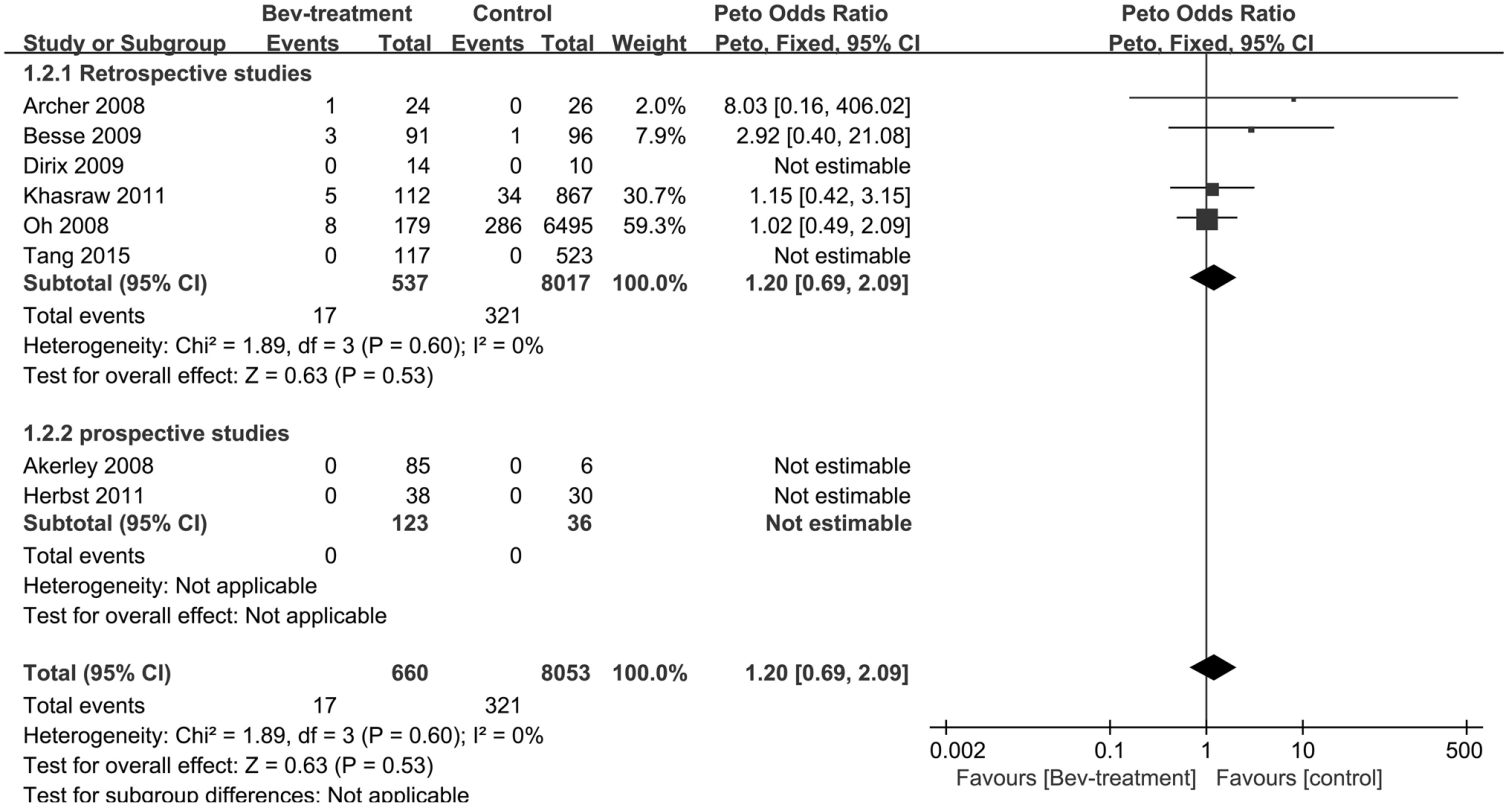
Incidence of ICH in bevacizumab versus control group

Eight studies, totaling 8713 patients and 338 events, provided data on ICH. In four studies, neither the experimental arm nor control arm had patients who developed ICH [25–27, 30], ICH occurred in the experimental arm, but not the control arm in one study [18], and the remaining three studies had ICH patients in both arms [21, 28, 29]. There was no heterogeneity across the eight studies included in the analysis (P = 0.60, I^2^ = 0%) despite definite differences, including primary tumor type, study design and bevacizumab dose. A fixed-effects model employing the Peto method was applied. Four studies in which no patients developed ICH in either the experimental arm or the control arm were excluded from meta-analysis by RevMan 5 during data processing, yielding results that were labeled “Not estimable” instead of producing an OR and CI. The pooled OR was 1.20, with 95% CI from 0.69 to 2.09 (P = 0.53). This result indicates that no significant increase in ICH was found in the bevacizumab-containing treatment arm compared with that in the control arm (Fig. 2).

Subgroup analysis was performed to assess the influence of study design on the OR for ICH. The analysis of six retrospective studies, with a total of 8554 patients and 338 events, revealed that ICH was not significantly elevated in the bevacizumab arm compared with that in the control arm (OR 1.20; 95% CI 0.69–2.09; P = 0.53). However, the analysis of the prospective study group failed because neither the bevacizumab arm nor control arm had patients who developed ICH in the two prospective studies, and RevMan 5 produced results that were labeled “Not estimable” instead of producing an OR and CI (Fig. 2).

### Publication bias

A funnel plot to evaluate publication bias requires at least ten studies to be included in the meta-analysis, otherwise, the test power will be too low to assess the symmetry of the funnel plot. However, the meta-analysis described here included only eight studies, and in four studies, neither the bevacizumab arm nor the control arm had patients who developed ICH. Therefore, we did not generate a funnel plot.

## Discussion

This is the first meta-analysis to evaluate the risk of ICH in BM patients with versus without bevacizumab treatment. We found no significant difference in risk of ICH in solid tumor patients with BM based on bevacizumab treatment. Furthermore, the results were consistent in subgroup analysis of the retrospective studies, although subgroup analysis of the prospective studies failed because not enough prospective studies were identified, and no patients developed ICH in the two prospective studies that were included.

Notably, four studies in which neither the experimental arm nor the control arm contained patients who developed ICH were excluded from this meta-analysis by RevMan 5 during data processing, and the results of this meta-analysis were derived from the remaining four studies. However, no difference in the risk of ICH was detected in the patients receiving the bevacizumab-containing versus non-bevacizumab-containing treatment in the four studies excluded from analysis, although the sample sizes of the bevacizumab arm and control arm in each excluded study are quite different. Thus, the results of the four excluded studies are consistent with the meta-analysis results of the remaining four studies. In addition, the same situation was observed in subgroup analysis.

In a phase II prospective noncomparative study (BRAIN, NCT00800202) investigating the efficacy and safety of bevacizumab in non-squamous NSCLC patients with asymptomatic untreated BM, only one of the 91 patients enrolled experienced an ICH event (grade 1) [31]. In another single-arm phase II trial (PASSPORT, AVF3752g) addressing bevacizumab safety in patients with non-squamous NSCLC and previously treated BM, no grade 2 CNS hemorrhages occurred [8]. The reported incidences of cerebral hemorrhage in patients with CNS metastases not exposed to bevacizumab range from 5 to 29% [21, 32–36]. Comparing these background ICH rates with those presented in the BRAIN and PASSPORT studies, there is no apparent increased risk of cerebral hemorrhage in bevacizumab-treated patients with CNS metastasis, although direct cross-trial comparisons should be viewed with caution. These results are consistent with our findings presented in this meta-analysis.

Clinical trials containing bevacizumab still routinely exclude patients with CNS metastasis due to concerns about the increased risk of ICH, arising partly from a single case in a phase I trial of bevacizumab, in which a 29-year-old patient with HCC experienced a fatal cerebral hemorrhage from a previously undiagnosed brain metastasis in 1997 [16]. However, the risk for spontaneous bleeding of CNS metastases varies with the histology of the tumor, with a < 1 or 5% chance of occurrence in lung or breast BM, respectively, compared with significantly higher rates for metastases derived from thyroid cancer, melanoma (40–50%), renal cell cancer (70%), choriocarcinoma or HCC [37]. CNS metastases from HCC have an inherent propensity to hemorrhage because patients with HCC are likely to have coagulation disorders due to liver dysfunction, resulting in ICH incidences up to 87.5%, independent of the type of therapy received [21, 38–41].

Our study has several limitations. First, patients with some factors, such as medications associated with bleeding (anticoagulants, etc.) [42], thrombocytopenia [42], tumor histology predisposing to bleeding [37], evidence of bleeding diathesis or coagulopathy [43], uncontrolled hypertension [44], or history of thrombotic or hemorrhagic disorders [44], are at risk of ICH intrinsically, and these factors are important stratified factors in evaluating whether bevacizumab increases the risk of ICH in patients with BM. However, limited stratified analysis of these factors was performed in this study because these factors were not reported in a portion of individual primary studies included in this meta-analysis. Second, the number of studies enrolled in this meta-analysis was small. Only eight studies met the eligibility criteria and were included in the analysis. Finally, not all the included studies were RCTs, which is the gold standard for clinical research and has less bias than other study designs.

In conclusion, this meta-analysis revealed that bevacizumab does not significantly increase the risk of ICH in solid tumor patients with BM, and it has provided evidence indicating that BM patients with a low incidence of ICH [21], such as those with advanced/metastatic breast cancer, NSCLC, renal and colorectal cancer, may not be generally excluded from bevacizumab therapy or trials. It will be important to validate these findings in RCTs with larger cohorts.
